# Ethylene: Management and breeding for postharvest quality in vegetable crops. A review

**DOI:** 10.3389/fpls.2022.968315

**Published:** 2022-11-14

**Authors:** Giacomo Cocetta, Alessandro Natalini

**Affiliations:** ^1^ Department of Agricultural and Environmental Sciences, Università degli Studi di Milano, Milano, Italy; ^2^ Council for Agricultural Research and Economics – Research Centre for Vegetable and Ornamental Crops, Monsampolo del Tronto, Italy

**Keywords:** fresh-cut, quality attributes, storage, fruits and vegetables, 1-MCP, new breeding techniques

## Abstract

Ethylene is a two-carbon gaseous plant growth regulator that involved in several important physiological events, including growth, development, ripening and senescence of fruits, vegetables, and ornamental crops. The hormone accelerates ripening of ethylene sensitive fruits, leafy greens and vegetables at micromolar concentrations, and its accumulation can led to fruit decay and waste during the postharvest stage. Several strategies of crops management and techniques of plant breeding have been attempted in the last decades to understand ethylene regulation pathways and ethylene-dependent biochemical and physiological processes, with the final aim to extend the produce shelf-life and improve the postharvest quality of fruits and vegetables. These investigation approaches involve the use of conventional and new breeding techniques, including precise genome-editing. This review paper aims to provide a relevant overview on the state of the art related to the use of modern breeding techniques focused on ethylene and ethylene-related metabolism, as well as on the possible postharvest technological applications for the postharvest management of ethylene-sensitive crops. An updated view and perspective on the implications of new breeding and management strategies to maintain the quality and the marketability of different crops during postharvest are given, with particular focus on: postharvest physiology (ethylene dependent) for mature and immature fruits and vegetables; postharvest quality management of vegetables: fresh and fresh cut products, focusing on the most important ethylene-dependent biochemical pathways; evolution of breeding technologies for facing old and new challenges in postharvest quality of vegetable crops: from conventional breeding and marker assisted selection to new breeding technologies focusing on transgenesis and gene editing. Examples of applied breeding techniques for model plants (tomato, zucchini and brocccoli) are given to elucidate ethylene metabolism, as well as beneficial and detrimental ethylene effects.

## Introduction: Evolution of breeding and ethylene management, for facing old and new challenges in postharvest

Ethylene (ET) was the first phytohormone to be discovered. It is an important regulator of several developmental and physiological processes: from seed dormancy to germination, fruit ripening, defence against biotic and abiotic stresses.

Due to its role in ripening, ET can lead to deleterious decay of vegetables, fruits, and ornamental crops. Thus, the biosynthesis of ET and ET-dependent pathways have been targeted in breeding activities, biotechnological and transgenic studies, in order to reduce waste and to extend the product shelf life (Pradhan et al., 2015; Houben and Van De Poel, 2019).

**Figure 1:**
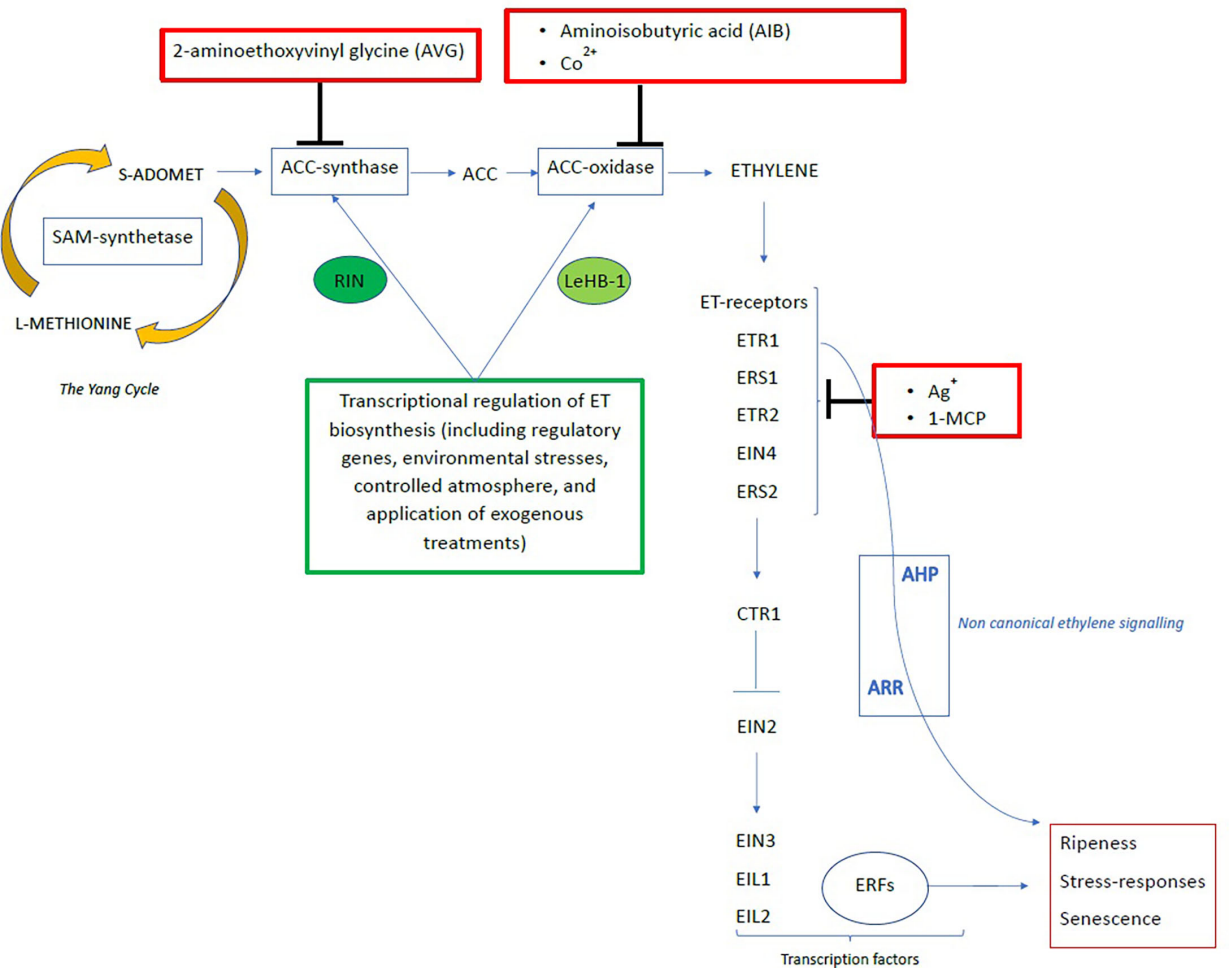
A simplified representation of the ethylene pathway including biosynthesis, signaling, and regulation (modif. Lin et al., 2009; Schaller and Binder, 2017; Binder, 2020).

ET biosynthesis is catalysed through the activities of ACS and ACO. At the onset of ripening, ET pathways are regulated by the “system 1” which is an auto-inhibitory phase with basal levels of the phytohormone and reduced ET response. Afterwards, the catalytic phase occurs in the “system 2” with a rapid increase of ET production due to a very high sensitivity to the phytohormone itself (Kumar et al., 2014). The hormone biosynthetic pathway consists of 2 steps: in the first reaction S-adenosylmethionine (SAM) is converted into 1-aminocyclopropane-1-carboxylic acid (ACC) by ACC-synthase (ACS); the second step is the conversion of ACC to ET by ACC-oxidase (ACO) (Houben and Van De Poel, 2019). Due to its involvement in several physiological processes, from development to postharvest physiology and storage, ET biosynthesis and signaling have been largely studied. In particular, to understand the complex elements that regulates ET signaling, is of great importance for controlling the ET action in order to prevent ET-induced postharvest losses. The so called “canonical” ET signaling pathway involves ET receptors (ETR1, ERS1, ETR2, EIN4, ERS2), the protein kinase CTR1, and EIN2 and the transcription factors EIN3, EIL1 and EIL2, which activate other transcription factors, including those belonging to the ERF family. Moreover, it has been proposed that another “non-canonical” signaling pathway, involving AHP and ARR regulation proteins (Binder, 2020) (**Figure 1**).

Some recent studies reported that, in order to avoid a dramatic poor nutrition, starvation, and related consequences, food production should double in the coming 50 years (Shipman et al., 2021). Many vegetables and fruits, which are characterized by a high nutritional value due to the content in vitamins and other bioactive compounds, are considered as perishable food commodities. Therefore, to preserve their postharvest quality is crucial for reducing wastage and to assure the maintenance of quality during the whole food production and distribution pipeline.

In this review, the Authors describe the importance and the role of ET in postharvest quality and provide an overview on the evolution of breeding technologies, in order to highlight the complex network of biological processes involving this phytohormone. This work aims to help in connecting the most important research areas related to ET to the technological applications that can provide a relevant extension of the product marketability, while preserving the quality and of produce.

## Ethylene-related postharvest management and physiological responses of crops

ET involvement in postharvest management of fruits, vegetables and ornamental crops has been widely studied and new insight in this topic indicate that this hormone plays very different and important roles in a specific manner, for different kind of products after harvest (Ebrahimi et al., 2022). Indeed, apart from its well-known involvement in the ripening and maturation processes in mature climacteric fruits, ET affects the marketability of immature and non-climacteric fruits, acts as signaling molecule in the development of certain postharvest physiological disorders (i.e. chilling injury). The increasing number of studies and discoveries are suggesting that the differentiation between climacteric and non- climacteric fruits, based on the ET role and involvement, are not always perfect and that ET-related mechanisms are not always clear, indicating that the phytohormone could have a ubiquitous role in fruit ripening (Paul et al., 2012). Moreover, ET affects the storability and the quality of several vegetables, including leafy greens, which are often commercialized as fresh cuts. This category of products has been largely studied for the effects of cutting operation on quality, and in this context, the wound-induced ET production is a crucial aspect to be considered.

### Climacteric fruits

A large amount of literature has been produced in the last years, regarding the effect of ET on climacteric fruit ripening, during cultivation and along the postharvest pipeline. Several works have been focused on tomato as it is considered as a model fruit for climacteric fruits (Alexander and Grierson, 2002), and, at the same time, it is one of the most widespread crops all over the world. For this reason, most of the research cited in this section, are related to this crop. For tomato as well as for other ET sensitive climacteric fruits, to find new strategies for preventing the product decay during storage and transportation is crucial to reduce product losses.

At a molecular level, to study the effect of different treatments on ET-receptors, can allow understanding how ET is affecting the storability and marketability of climacteric fruits. In a recent study, different ET receptors were investigated at a transcriptomic level in tomato during ripening as well as off vine. This approach included the use of 1-methylciclopropene (1-MCP) for understanding the regulation of ripening time and the role of ET action and for underlying those among the genes and responsive elements, were the most sensitive and relevant in deciphering the ET mediate responses in different conditions (Mata et al., 2018). Latest discoveries suggested the presence of an interplay between ET and methyl jasmonate (MJ) metabolism, in regulating ripening in climacteric fruits. MJ is a plant hormone largely studied in several plant species. In a recent publication, it has been showed that tomato *ETR3, ETR4, ETR6* and *ETR7*genes, were stimulated by MJ application in tomato and ripening was accelerated during postharvest (Tao et al., 2021).

Other plant hormones have been proven to interact with ET metabolism, regulating fruit ripening. Salicylic acid (SA) exogenous application has been showed to delaying ripening in different species. Moreover, a recent study showed that tomato lines having high levels of endogenous SA, produced less ET and had a longer shelf life (Changwal et al., 2021). In the case of another important climacteric crop, avocado, it has been shown that the evolution of postharvest ripening is not only depending on the ET action, but it is regulated by a complex interplay of different hormones including auxin, jasmonates, GA and ABA (Vincent et al., 2020).

Exogenous application of melatonin increased ethylene production and up-regulated the 1-aminocyclopropane-1-carboxylic acid (ACC) synthase gene expression. Also, the ethylene receptor genes, NR and ETR4, and the transducing elements, EIL1, EIL3 and ERF2, were upregulated by melatonin application (Arnao and Hernández-Ruiz, 2018).

These results would suggest the possibility of applying specific hormones exogenously, for modulating postharvest ripening processes and prolonging shelf-life. Further studies should be conducted, testing the timing, mode and dosage of application for different crop species.

Another aspect that has been investigated in tomato is the correlation between ET action and membrane catabolic enzymes such as phospholipases (Natalini et al., 2017) and lipoxygenase (Velázquez-López et al., 2020). By silencing the main isoform of lipoxygenase encoding gene (*TomloxB*) it was possible to conclude that lipoxygenase could be a regulator of ET synthesis through the activity of the enzyme ACO. The Authors suggested that this discover could be useful for improving the shelf-life extension and the postharvest management of tomato (Velázquez-López et al., 2020).

Several technological approaches can be used for counteracting and reducing the negative effects of ET exposure during the postharvest management of several kinds of climacteric fruits. Heat treatments have been suggested as an efficient strategy to prolong the tomato storability through the induction of DNA methylation as response of ET signaling mechanisms (Pu et al., 2020).

Other strategies have been traditionally studied and applied, and include the use of controlled and modified atmosphere, packaging (with or without atmosphere modification) (Hu et al., 2019). The gas atmosphere modification and control are effective in extending the shelf-life and storability because, by increasing the levels of CO_2_ and reducing O_2_, it is possible to slow down the respiration rate and inhibit the ET biosynthesis. The combination of atmosphere modification with packaging has been largely studied and applied, however the increasing concern related to the use of plastic, should be considered when evaluating the sustainability of these technological solutions.

ET absorbers and scavengers (Álvarez-Hernández et al., 2018; De Bruijn et al., 2020; Wei et al., 2021), ventilation systems, ozone, active oxygen and 1-MPC have been used to extend the shelf-life (Wills, 2021). About 1-MCP, there is large evidence of its efficacy in climacteric fruits and new form of applications have been studied, including the possibility to incapsulate the gaseous molecules into microbubbles, to be delivered in a more precise manner, as showed in the case of melon (Le Nguyen et al., 2019).

Vacuum Ultraviolet Photolysis is novel technique that has been proposed for limiting the negative impact of ET, based on the generation of highly reactive radicals that can oxidise ET to carbon dioxide and water while simultaneously inactivating pathogenic microorganisms. This technique has been tested on several products, including fruits and vegetables (Mabusela et al., 2022).

Another recent study showed that treatments with ethanol were able to inhibit the ripening of tomato fruit, while stimulating ET production (Suzuki and Nagata, 2019). This controversial finding is very interesting and suggest further investigations on this topic.

### Non-climacteric fruits

Several plant-based foods are physiologically immature and non—climacteric fruits. This category, including zucchini, among others, is generally considered less perishable compared to those fruits that are commercialized and consumed at full ripening stage, but their postharvest management still presents some potential risks and issues, which in some cases involves ET. Several studies showed that ET and ET-related genes are involved in the development of chilling injury (CI) in some fruits during cold storage, including zucchini. Zucchinis are commonly stored at low temperature (4°C) and in this phase the fruits can show the detrimental symptoms of CI.

Several studies showed that CI symptoms are accompanied by an increment in ET production in zucchinis subjected to cold storage and then kept at 20°C. A relationship between chilling-induced ET production and chilling sensitivity in different cultivars of zucchini, allowed demonstrating the involvement of ET in CI development, although ET is not considered as the only factor inducing CI. During CI, a burst in reactive oxygen species (ROS) production is usually observed which leads to the membrane’s degradation, so it is possible that the increment in ET production is related to the increasing oxidative stress (Megías et al., 2014). A similar role has been suggested also for summer squash (Megías et al., 2017). The role of oxidative stress in CI development in immature fruits has been well described in a recent review paper (see Valenzuela et al., 2017 and references herein). Further studies demonstrated a role for ET in inducing CI symptoms, by using the ET-inhibitor 1-MCP, for blocking ET biosynthesis and perception. The application of 1-MCP, reduced the CI symptoms in CI sensitive zucchini cultivars and induced the downregulation of the genes involved in the ET biosynthesis and perception (Megías et al., 2016). Moreover, the use of shrink-wrapping packaging, has been proposed as an effective strategy to mitigate the negative effects of ET-mediated CI (Megías et al., 2015).

A recent study aimed to compare the ET receptors and related protein in climacteric and non-climacteric fruits (Chen et al., 2018). The results obtained showed that both categories share many of the ET perception and signaling pathways. However, non-climacteric fruits (grape and citrus) had a lower number of ethylene-responsive factors. ET receptors (i.e. *FaEtr1* and *FaErs1*) have been found to be active also during strawberry ripening, suggesting that the hormone can in fact play an important role in non-climacteric fruits (Chen et al., 2018).

Considering these aspects, it is interesting to notice how 1-MCP could be used for both research and commercial/practical purposes. In fact, by inhibiting the ET applying 1-MCP it is possible to understand those physiological and metabolic events that are at least partially depending on the ET action, also in non-climacteric fruits and at the same time it is possible to use it as a powerful tool in order to extend the commercial life of several crops, including non-climacteric fruits. 1-MCP can act by delaying senescence and senescence-associated disorders (e.g. degreening, color changes) during postharvest, as mentioned earlier in this paragraph. Furthermore 1-MCP can limit the development of certain physiological disorders, including chilling injury. It has been also found that the effect of 1-MCP on respiration and decay development in non-climacteric fruit can vary depending on the considered crop (for a detailed review on this topic see: Li et al., 2016).

### Vegetables

Vegetables-based foods include a wide variety of species and plant organs (leaves, stems, flowers, tubers, roots) characterized by different sensitivity to ET action during postharvest and by a different management during transportation, storage, and retail.

Among vegetables, different species belonging to the *Brassicaceae* family are particularly sensitive to ET, which can induce yellowing and led to a rapid produce degradation after harvest and during storage

Broccoli (*Brassica oleracea* L. var. Italica) florets are particularly sensitive to ET action and novel strategies are needed to control its negative effects. An efficient approach consists in the use of ET absorbers, also coupled with packaging. This combined approach, based on ET removal and passive modified atmosphere packaging was proven to be effective in extending the shelf life of this perishable vegetable product (Esturk et al., 2014). A novel approach, based on gene expression analyses, was applied for understanding the molecular responses in which ET was involved during storage at different temperatures in cabbage, broccoli, and kale (Ahlawat and Liu, 2021). This research showed that ET signaling genes were upregulated at room temperature storage (25°C) in broccoli and kale, whilst lower temperature (4°C) reduces the gene expression (as reported more in detail in the following section). The same study showed that low temperature selectively reduces the expression of senescence associate genes (SAGs) and ET receptors. These finds suggest the important role of temperature in ET management during storage and shelf life. Moreover, in cabbage, Miret et al. (2018) demonstrated that abscisic acid treatments reduce the ET biosynthesis and its accumulation inside the storage package by half, whilst the water and chlorophyll contents were not affected. In *Brassica*, during postharvest, ET and cytokinin treatments affected the postharvest quality of broccoli heads by altering the lipoxygenase activity which is responsible of degreening. Indeed, cytokinin applications delayed the physiological increase of *BoLOX1* expression and lipoxygenase activity whereas ET accelerated both processes (Gomez-Lobato et al., 2012a). Furthermore, the use of the ET antagonist (1-MCP) reduced chlorophyll degradation and the expression of genes associated to chlorophyll catabolism with different actions on several chlorophyllases (*BoCLH1* and *BoCLH2*) (Gómez-Lobato et al., 2012b).Celery is a vegetable that can be consumed fresh or commercialized as ready to eat product, after a minimal processing. In this context, cutting operation triggers the production of ET which, in turn can induce an accelerated senescence processes and finally led to product decay. Applications of 1-MCP have been proposed as an efficient strategy to prolong the shelf life of fresh-cut celery petioles. The treatment decreased discoloration, soft rots, yeast counts and improved overall quality maintenance. These results suggested a potential role for ET in compromising celery postharvest quality (Massolo et al., 2019). The involvement of the phytohormone in wounded-induced senescence is a very important and well-studied phenomenon, affecting the storability of several leafy vegetables, commercialized as fresh-cuts, especially in lettuce (Iakimova and Woltering, 2018; Ripoll et al., 2019). Wounding induces a cascade of reactions involving ET, SA and MJ, and in turn it can trigger secondary metabolite biosynthesis, as shown in a recent study conducted on broccoli (Guan et al., 2021).

## The role of ethylene in postharvest quality of fresh and fresh cut products

All the conditions and physiological events described in the previous paragraph, are among the crucial players which affect the commercial success of a produce during postharvest. The extension of storage time and marketability are very important issues, but at the same time, quality must be maintained as high as possible from the field to the final consumer. Thus, for all the strategies and technologies used for limiting ET biosynthesis and accumulation, the effect on the product quality and the consumers acceptability should be taken into consideration. Quality is the result of several traits including visual appearance, colour, texture, nutritional and nutraceutical value, aroma, flavour and tase, among others. Many of these characteristics can be affected by ET after harvest (Saltveit, 1999) and along the commercial life of a produce and different treatments and technological solutions can be applied in postharvest for limiting ET accumulation and effects while improving and maintaining the quality.

Melatonin has been studied as a potential treatment for delaying senescence and prolong the shelf-life by affecting ET metabolism and action. Melatonin treatments were effective in delaying senescence and limiting the ET action while improving quality traits in broccoli florets. In fact, melatonin-treated broccoli showed a better colour maintenance during storage and a higher content in chlorophyll (Hu et al., 2022).

In a recent study, hydrogen sulphide, was effective in delaying ripening and senescence of tomato fruits during the storage period. The treatment allowed maintaining higher levels of chlorophyll, starch, soluble proteins and ascorbic acid, compared to untreated control fruits. The Authors suggested that hydrogen sulphide could act as a signal molecule, responsible for the ET repression during postharvest (Yao et al., 2020). Also, selenium treatments were effective in repressing the expression of ET biosynthetic genes, and in stimulating the enzymatic antioxidant systems in tomato, while prolonging the shelf life (Zhu et al., 2017). Another widely used product, 1-MCP can have different effects on the quality attributes of climacteric fruits like tomatoes (Poyesh et al., 2018; Zou et al., 2018; Taye et al., 2019) pears (Cocetta et al., 2016), apples (Kim et al., 2018) and bananas (Satekge and Magwaza, 2020). On the other hand, it has been reported to have just slight effects on internal fruit quality of non-climacteric fruits (Li et al., 2016).

In case of the highly perishable vegetables broccoli, the application of amino acids was effective in decreasing ET production, delaying discoloration and determining highest antioxidant capacity (Sohail et al., 2021a; Sohail et al., 2021b).

Modified atmosphere storage was applied as an effective strategy to maintain quality attributes in whole and fresh cut romaine lettuce during storage. This approach allowed modulating the internal gas composition of the package, affecting ET production, with an effect on sensory quality and marketability, with some differences depending on the kind of product (whole vs. fresh-cut) and storage temperature (Choi et al., 2021). Postharvest commercial management of crops, from field to fork, implies some difficulties during the transportation and in case of ripe fruits, the potential risk of negatively affect the quality is high. Tomatoes can be easily subjected to mechanical damages which in turn lead to quality losses. A recent study showed that even imperceptible compression of tomato fruits, which could occur during different phases of the postharvest pipeline, can significantly affect the product quality and marketability. These effects involve the action of ET and include an increase in the hormone production, a delay in colour development, a transient activation of oxylipin pathway enzymes activity and a decrement in the emission of volatile compounds linked to sweet and fruity sensory traits (Spricigo et al., 2021).

## Evolution of breeding technologies for postharvest quality

### From conventional breeding to transgenesis for ET

As previously described, ET controls a relevant range of biochemical, physiological, and molecular processes that affect postharvest storage. Alteration in the ET biosynthesis and perception mechanisms can have positive effects, by prolonging the product shelf-life, but further efforts can help emphasizing the postharvest quality of crops (Shipman et al., 2021).

In this section, the Authors will refer to some representative crop models, chosen among each one of the categories described in the previous section, namely tomato, broccoli and zucchini for climacteric fruits, non-climacteric fruits and vegetables, respectively.

### Crop model: Tomato

Tomato is often considered the reference crop for fleshy fruit development and its ripening is controlled by ET dependent and independent pathways (Passam et al., 2007).

To investigate ET biochemical and molecular pathways, most of the studies related to molecular assisted selection (MAS) in conventional breeding are based on crosses carried out between cultivated tomato or elite lines and related species, or most commonly, mutants. Indeed, tomato is a self-pollinating species and can also easily hybridize with wild relative species belonging to *Solanum* genus, thus allowing gene introgression. The availability of short life cycle genotypes (i.e. Microtom) can further optimize the breeding efforts, being a model transformation system for functional genomics analyses (Dan et al., 2006).

Several qualitative traits related to ET dependent ripening, are determined by complex regulatory networks, under polygenic control and quantitative inheritance. The genome regions which include the genes associated with a polygenic trait are called quantitative trait loci (QTLs). The efficiency of the introgression of a quantitative trait locus (QTL) depends on the stability of QTL expression (Chaïb et al., 2006). Generally, tomato fruit traits are quantitatively inherited and several QTLs associated with fruit development, size, shape, colour, ripening, sensorial quality and yield have been identified (Van Der Knaap and Tanksley, 2003). Since the last two decades, transgenic transformation has been applied to tomato to elucidate ET and its biochemical and metabolomic pathways. One of the first transformation used the gene of bacteriophage T3 that encodes for the enzyme S-adenosylmethionine hydrolase (SAMase) (Good et al., 1994). Houben and Van De Poel (2019) generated transgenic tomato plants with an altered capacity to synthesize ET. Interestingly, to restrict the presence of SAMase to the ripening fruit, the researchers used the tomato E8 gene as promoter. This gene regulates SAMase expression at specific stage of ripening. Indeed, SAMase expression was regulated only during the breaker and orange stage of ripening, whilst its expression was stopped at full ripe stage of maturity.

Another example of transgenic transformation applied to investigate the ET pathway, accompanied by a metabolomic approach, has been presented by Sobolev et al. (2014). The Authors generated a homozygous transgenic tomato genotype (2AS-AS) that showed reduced ET production and longer shelf-life. The genotype showed an impaired expression of ACS gene by antisense RNA. Furthermore, a double transgenic hybrid (2AS-AS × 579HO) was developed through a cross between 2AS-AS and 579HO, which resulted in significantly higher ET production compared to the wild-type or 2AS-AS fruit (Sobolev et al., 2014).

Another strategy was based on the release of commercial tomato varieties developed with recombinant DNA technology. The first attempt was the variety Flavr Savr, approved by the Food and Drug Administration (Silver Spring, MD, USA) in 1994. Flavr Savr showed a remarkable shelf-life due to the introduction of the antisense polygalacturonase (PG) gene, an ET dependent enzyme involved in the fruit softening process, but its taste was poor and the variety was discontinued (Krieger et al., 2008).

The relationship between ET biosynthesis and the induction of the climacteric peak of respiration during fruit ripening, have been investigated in tomato over the past 50 years (Goodenough, 1986; Chaves Soares and Mello-Farias, 2006), but the molecular and metabolomic mechanisms involved are still under investigation. In addition to transgenic technology, gene transfer by genome editing methodologies can lead to the integration of the transgene/cisgene, in order to have a more predictable expression (Cardi and Neal Stewart, 2016). Nowadays, the most applied site-specific genome editing technology is the type II clustered regularly interspaced short palindromic repeat (CRISPR)/Cas9 (CRISPR-associated) firstly identified in *Streptococcus pyogenes* and applied for the first time in 2013 in five different plant species as reported by Bortesi and Fischer (2015).

In climacteric fruits, ET synthesis, regulation, and perception lead to the transcription of ripening-regulated genes that affect quality. The ET signaling include several components: various ET receptors in the membrane of the endoplasmic reticulum, the protein kinase termed constitutive triple response 1 (CTR1), an endoplasmic reticulum-localized transmembrane protein of unknown biochemical activity termed ET-insensitive 2 (EIN2), TFs such as EIN3, EIN3like and ET response factors (ERFs) (Binder, 2020) (**Figure 1**). Also, several studies revealed the presence of a crosstalk occuring between ET and other phytohormones including auxins and ABA. Kumar and co-authors reported the upregulation of both ACS and ACO genes induced by auxins.

During ripening, relevant changes occur in texture, colour and aroma which are regulated from ET together with transcription factors (TFs) and their downstream effector genes. Extensive reviews deeply investigate TFs as reported in Giovannoni et al. (2017) and Wang et al. (2019a). ET signal pathway is a complex transduction network that leads to the activation of downstream transcriptional regulators ERFs (Gao et al., 2020) (**Figure 1**). ERF proteins are encoded by one of the most consistent families of plant TFs, among which, the APETALA2/ethylene response factor (AP2/ERF) is one of the most important during ripening and in the regulation of ET-responsive genes (Fenn and Giovannoni, 2021). In tomato, other families of TFs involved in ET response and fruit ripening are the *RIN-MADS, CLEAR NON-RIPENING, TAGL1* and *LeHB-1* genes which encode positive regulators of ripening. Contrastingly, AP2/ERF negatively regulates ET biosynthesis but it is a positive regulator of other fruit ripening phenomena such as chlorophyll degradation and carotenoid biosynthesis (Wang et al., 2019a). Indeed, RNAi repression of AP2/ERF results in fruits that over-produce ET, hasten fruit ripening and alter carotenoid pathway and accumulation (Chung et al., 2010).

Spontaneous tomato mutants have been deeply used to investigate the functions of ET-dependent genes through the observation of the ripening phenotype and mapping the genes underlying the mutation (Wang et al., 2020). The most important monogenic tomato mutants include ripening-inhibitor (*rin*), *nonripening* (nor), *colorless nonripening* (*Cnr*), *green-ripe* (*Gr*), *green flesh* (*gf*), *high pigmnet1* (*hp1*), *high pigment2* (*hp2*) and *never ripe* (*Nr*) (Osorio et al., 2020). Furthermore, *rin* and *nor* mutations have been used also for commercial purposes and for developing parental lines showing a delay in the ripening and in textural deterioration. This strategy helped to develop new varieties characterized by a longer shelf-life and by a lower rate of produce deterioration/losses.

Tomato mutants are available for research purposes at various institutions, e.g., C.M. Rick Tomato Genetic Resource Center (http://tgrc.ucdavis.edu/); Hebrew University (http://zamir.sgn.cornell.edu/mutants/) (Giovannoni, 2004).

As described in Shipman et al. (2021), in several climacteric fruits, the genetic suppression or silencing of ACO and ACS lead to a reduction of ET biosynthesis, which in turns lead to a delay in the ripening process.

A milestone review was published in 2002 and provided important information related to the understanding of the specific isoforms of ACS and ACO and their role in regulating ET biosynthesis during ripening. It also provided important information about the role of ET receptors (Alexander and Grierson, 2002). The *never ripe* (*Nr*) mutant and the *ripening inhibitor* (*rin*) mutant have been used to identify which of the ACS genes among *LeACS1A*, *LeACS2*, *LeACS4*, *LeACS6* (at least 12 forms have been found in the tomato genome) are ET regulated (Seymour et al., 2013). The *Nr* fruit ripening locus is responsible for a mutation in the ET -binding domain of the *Nr* ET receptor, which alter the signaling and perception for the phytohormone (Alexander and Grierson, 2002; Giovannoni, 2004). On the other hand, the *rin* mutant do not exhibit autocatalytic ET production and shows an altered ET signal downstream due to a genomic DNA deletion, resulting in the fusion of two truncated transcription factors, RIN and MC in the RIN transcription factor (Alexander and Grierson, 2002; Li et al., 2018; Gao et al., 2019). *Nr* and *rin* mutant fruits have shown that *LeACS2* expression requires ET whilst *LeACS1A* and *LeACS4* revealed only a delay in the expression in *Nr*. These evidences suggested that ET is not responsible for regulation of these genes and both *LeACS1A* and *LeACS4* are involved in the production of system 1 ET (Barry et al., 2000; Alexander and Grierson, 2002). In *rin* mutant, as well as in mature green wild-type, all ACS genes revealed the same patterns of expression, but did not show any ripening-related changes of expression (Barry et al., 2000).

### Crop model: Broccoli

The *Brassicaceae* family is derived from the included genus *Brassica* and several important crops belong to this taxonomic group including broccoli, cauliflowers, Brussels sprouts, cabbage, kale, mustard (greens), and collards. This genus is widely distributed world-wide, except for Antarctica and include leaf and root vegetables, as well as oilseed crops. In *Brassica* genus, researches on ET have been conducted to elucidate the most relevant biochemical and physiological pathways, while only a few investigations have been conducted for breeding and molecular purposes compared to other crops like tomatoes. For this reason the hormone signaling cascade pathway in these species is still not clear (Ahlawat and Liu, 2021). Recently, several gene families named senescence-associated genes (SAGs) and two families of transcription factors named: *NAC* (*NAM*, *ATAF* and *CUC*) and *WRKY* which are involved in the senescence process, have been detected in *Arabidopsis thaliana* (Guo and Ecker, 2004; Balazadeh et al., 2011; Rauf et al., 2013; Kim et al., 2014) and investigated in *Brassicaceae* such as cabbage, kale and broccoli (Ahlawat and Liu, 2021). In this novel research, the products were stored at room temperature (25°C) and at low temperature (4°C). At room temperature, the expression of the SAGs genes *ORE15*, *SAG12* and *NAC29*, increased, while low temperature reduced the expression levels for *ORE15* and *SAG12*, with differences among the crops under investigations. Furthermore, also the transcript of ET receptors as well as those of genes involved in the ET biosynthesis, were affected by the temperature in cabbage, suggesting different molecular events among *Brassica* vegetables during storage and shelf life.

ET affect floret yellowing in broccoli as demonstrated by Tian et al. (1994) with the application of both ET and propylene (an ET functional analogue molecule). Indeed, both molecules stimulated respiration and ET production as well as yellowing, measured as chlorophyll and hue-angle decline on broccoli heads (Tian et al., 1994). Through directed mutagenesis, a mutant broccoli ET-response sensor (*ers*) gene was obtained, and two plasmids were constructed obtaining two transgenic lines (Chen et al., 2004). Transgenic broccoli showed ET insensitivity, a delaying in leaves senescence as well as a retard of 1-2 days in head yellowing, compared to non-mutant lines (Chen et al., 2004).

Also, *Brassica oleracea ACC synthase* and *ACC oxidase* were co-transformed into a double haploid calabrese broccoli (GDDH33) to elucidate the role of ACS and ACO into ET biosynthesis in *Brassica* genus (Higgins et al., 2006). The ACS-engineered plants showed a reduction in the postharvest ET peak heigh and a delay in chlorophyll degradation. Similarly, at 20°C, buds generated from T0 plants transformed for ACO, produced less ET compared to the untransformed plants (Higgins et al., 2006).

Gapper et al. (2002) engineered broccoli for ACO gene through antisense transformation. Transgenic broccoli harboring the antisense ACO showed a block into ET biosynthesis which, in turns, increased the transformation rate because the inhibition of ET biosynthesis increased the biosynthesis of shoot regenerative enhancing compounds (the polyamines) (Gapper et al., 2002).

### Crop model: Zucchini

Zucchini (*Cucurbita pepo*) is one of the most important crops in the *Cucurbitaceae* family due to its world-wide distribution (open field and off-season greenhouse productions) and its economic value (Nee, 1990). ET has been deeply investigated in zucchini for breeding purposes related to parthenocarpy (Martínez et al., 2013; Martínez et al., 2014; Sharif and Qi, 2022) and less for postharvest quality.

Interestingly, Garcia et al. (2017) developed a mutant collection of approximatively 3800 M2 families using MUC-16 as progenitor genetic background (WT) and ethyl methane sulfonate (EMS) as mutant agent. A consistent mutant phenotyping showed that mutations conferring ET insensitivity or altered ET sensitivity showed alter seedling germination, sex determination, sex expression parthenocarpy as well as fruit set (García et al., 2017).

In zucchini, ET has been found to be involved in chilling injury (CI) sensitivity, indeed more tolerant varieties showed a lower ET production at low temperatures (Megías et al., 2017). In a recent study (Megías et al., 2017) an ET-insensitive mutant *etr2b* and its WT have been compared in terms of ET production, respiration rate and oxidative stress. The research demonstrated that *etr2b* mutation (mutation on the coding region for ET receptor gene *CpETR2B*) could be of interest for future breeding programmes. After a storage period at 4°C, the homozygous mutant zucchini *etr2b/etr2b*, showed a lower level of CI, associated to a lower cold-induced ET production, compared to WT zucchini. Mutant fruits showed a lower hydrogen peroxide accumulation compared to WT fruits, while at 4°C, the respiration rate did not change significantly (Megías et al., 2017). Furthermore, the use of ET-insensitive zucchini mutant *etr2b* allowed demonstrating the involvement of ET in the development of cold-induced postharvest oxidative damage (García et al., 2020). As proposed by the Authors, cold-induced ET production can be used as a marker for individuating cold-tolerant zucchini genotypes.

In Spanish genotypes, the different expression of ACS and ACO genes has been investigated during cold storage at different temperatures (4°C, 12°C and 20°C) for 7 days (Megías et al., 2012). In the more CI tolerant genotypes, lower expression levels have been detected for *CpACS* and *CpACO*, while the most susceptible genotypes revealed a chilling-induced ET peak at 4°C, after the appearance of visible symptoms for CI (Megías et al., 2012).

Interestingly, a genotyping by sequencing analysis of an interspecific population derived from a cross zucchini (*C. pepo* ssp. *pepo*) x Scallop (*C. pepo* ssp. *ovifera*) generated more than 7000 thousand SNP markers and an high density linkage map covering 2,817.6 cM of the whole genome. Furthermore, 48 QTLs have been identified for vine, flowering and fruit quality based on a three environmental analysis and different TF involved in the ET metabolism have been identified in the DFeF_9 region, closed to other genes controlling the flowering process (Montero-Pau et al., 2017).

For breeding purposes, the availability of the *C. pepo* genome annotation at: https://cucurbigene.upv.es is a useful tool for the identification of QTLs underlying specific processes and to constitute new varieties.

### New breeding techniques (NBT): CRISPR/Cas9 leads to a re-evaluation of ET signal and TFs

The new findings related to the use of ethylene mutants obtained using CRISP/Cas9 approach are promising, also concerning the importance of using these mutants for increasing the knowledge and understanding of ethylene-dependent pathways, which play a key role, also during postharvest, and to develop, in the near future, novel strategies for improving crops quality and shelf life extension.

In tomato, CRISPR technology has been applied to the regulatory proteins CNR and NOR and to TFs: AP2a, FUL1, FUL2 with targeted deletion or substitution (Ito et al., 2015; Yu et al., 2017; Gao et al., 2019; Wang et al., 2019b). In absence of available spontaneous mutants, CRISPR/Cas9 offer a very efficient techniques to create the null mutants for mutagenesis. Previously, RNA interference (RNAi), or Virus-induced Gene Silencing (VIGS) were used to evaluate gene-function, but they revealed lack of specificity for the targeted genes, or incomplete suppression of expression (Wang et al., 2019b). Wang and co-authors applied CRISPR/Cas9 technology to further investigate on the role of APETALA2a (AP2a), NON-RIPENING (NOR) and FRUITFULL (FUL1/TDR4 and FUL2/MBP7) TFs on natural mutants. The use of gene-editing technology to knock out the encoding genes provided further insights on the functions of the TFs. The edited lines: *ap2a-cr1* and *ap2a-cr2* obtained with deletions in the first 9 exons of ap2 domain, showed higher ethylene production compared to the natural *nor* mutant line accompanied to a faster ripening, which confirm the negative role of AP2a in ET production. Similarly to previous results obtained with RNAi phenotypes (Karlova et al., 2011), these edited lines showed orange/brown fruit colour due to an incomplete chlorophyll degradation and to an impairment in the lycopene biosynthesis. Furthermore, the *nor* null mutant revealed a milder phenotype compared to the spontaneous *nor* mutant in which a dominant negative allele act with interlocus interaction instead of classical intralocus interaction. Moreover, null mutants providing null alleles FUL1 and FUL2 revealed partially redundant functions in fruit ripening and additional role for FUL2 in early fruit development (Wang et al., 2019a).

In the recent years, CRISPR/Cas9 knockout and RNAi silencing of *rin* in the wild-type tomato only partially restored the non ripening phenotype, because did not repress the initiation of ripening and the mutant fruits showed a moderate red colouration. The inactivation of the *rin* mutant allele partially restored the induction of ripening (Ito et al., 2017; Lü et al., 2018). Knockout or RNAi silencing of the chimeric RIN-MC mutant protein in *rin* background partially restored ripening. The fruits turned to a weak red colouration and these results suggest that *rin* is re-evaluated as a gain-of function mutant (Li et al., 2018; Gao et al., 2019). In other terms, *rin* phenotype is caused by the production of a fusion protein RIN-MC, instead of the loss of function of MADS-RIN (Ito et al., 2017; Wang et al., 2019a).

Interestingly, Gao et al. (2019) applied CRISPR/Cas9 to generate multiple knockout mutations in the gene loci for *cnr* and *nor*. The edited tomatoes revealed: i) a delay in ripening for the CNR CRISPR lines; ii) the NOR lines showed partial non-ripening phenotypes like the RIN CRISPR/Cas9 mutants; iii) both edited lines were different from the natural mutant NOR phenotypes.

Interestingly, Wang et al. (2019b) used CRISPR/Cas9 mutagenesis to knock out the encoding genes of *AP2a*, *NOR*, *FUL1* and *FUL2*. As reported by Martin-Pizarro and Posé (2018), the use of a gene editing approach, such CRISPR/Cas9, edited ET mutants could have practical use in tomato breeding for postharvest. In the past, RIN/rin hybrid plants have been used to reduce ET biosynthesis (due to incomplete ripening) and increase shelf life combined with poor flavor and reduced nutritional level. Nowadays, with alternative knockout alleles for ET biosynthesis, it is possible to improve fruit shelf life without any detrimental to organoleptic and nutritional quality (Martin-Pizarro and Posé (2018).

Beside CRISPR/Cas9 approaches to investigate the role of ET in null mutants for ripening, this NBT has been used to produce mutants with enhancements in postharvest quality parameters related to ET. Uluisk et al. (2016) generated tomato mutants for pectate lyase, an ET dependent enzyme involved in fruit softening, which showed an improvement in fruit texture and postharvest quality without altering color and soluble solids content. Other CRISPR/Cas9 based mutants for texture related genes have been generated for the following pectin-degrading enzyme: β-galactanase, polygalacturonase and pectate lyase. The investigation on pectin localization, distribution and their solubility in the CRISPR/Cas9 mutant fruits, revealed that only mutations for pectate lyase resulted in firmner fruits compared to the WT (Wang et al., 2019b).

To our knowledge, there are no published work on the application of CRISPR/Cas9-induced mutations for ET in breeding purposes related to postharvest quality in Brassica and zucchini.

These studies provided new evidence that CRISPR/Cas9-induced mutations in the tomato ripening transcription factor failed to abolish ripening, suggesting that the ripening transcriptional regulatory network has partial “back-up” properties with several key points of control. Only precise gene editing tools, as CRISPR/Cas9 applications, furnish more insights compared to previous techniques (e.g. RNAi) because they give the possibility to deeply understand gene function even between similar paralogs (es. FUL1 and FUL2), leading to a re-evaluations of the ET pathway and related fruit ripening and to explore the molecular basis of ET which are still unrevealing for important crops like broccoli and zucchini.

Therefore, deeper insights on ET pathways could fasten breeding times to develop improved varieties.

## Conclusions

ET has a crucial role in determining the quality and the storability of fruits and vegetables. For this reason, it has been largely studied under biochemical, technological, molecular and metabolomic approaches.

In this review the Authors aimed to give a general overview about to the main aspects related to the physiological and metabolic mechanisms behind the ET-dependent postharvest management of fruits and vegetables. The strategies to be applied should adapt to the specific characteristic of a crop and can space from long storage in cold rooms (which is widely used for apples and pears) to short storage at the retailer with or without the use of packaging and modified atmosphere (more used in the case of vegetables and fresh cuts). Moreover, chemical, and physical treatments can be already used or will be further explored and studied, in order to expand the array of tools to be used for prolonging the products commercial life.

From a general revision of the current literature, it appears clear that quality is a key point in postharvest management, and that the control of ET is a pivotal factor for the maintenance of high quality along the postharvest pipeline.

In this review paper, the authors emphasized the CRISPR/Cas9 method for its flexibility in gene editing that could lead the research of the present and recent future.

This technology represents a tool to re-evaluate previously genetic models generated from traditional genetic studies and developed solely based on single species studies. Furthermore, complex and important biological processes such as ripening are often controlled by highly redundant transcriptional network with inputs from multiple epigenome levels (Wang et al., 2019a).

For example, the use of CRISPR/Cas9 to investigate TF may improve our understanding of the molecular regulation of ET response in vegetables. These findings could allow the development of novel strategies for the control of ET biosynthesis and to modulate the ripening process at the rate and according to the needs of the supply chain, while maintaining quality (Shipman et al., 2021).

## Author contributions

Original idea: AN; Writing—original draft preparation, GC and AN equally contributed; writing—review and editing, GC and AN equally contributed. All authors have read and agreed to the published version of the manuscript.

## Conflict of interest

The authors declare that the research was conducted in the absence of any commercial or financial relationships that could be construed as a potential conflict of interest.

## Publisher’s note

All claims expressed in this article are solely those of the authors and do not necessarily represent those of their affiliated organizations, or those of the publisher, the editors and the reviewers. Any product that may be evaluated in this article, or claim that may be made by its manufacturer, is not guaranteed or endorsed by the publisher.
